# Deep Learning-Based Indoor Localization Using Multi-View BLE Signal

**DOI:** 10.3390/s22072759

**Published:** 2022-04-02

**Authors:** Aristotelis Koutris, Theodoros Siozos, Yannis Kopsinis, Aggelos Pikrakis, Timon Merk, Matthias Mahlig, Stylianos Papaharalabos, Peter Karlsson

**Affiliations:** 1 Libra AI Technologies, 11854 Athens, Greece; aris.koutris@libramli.ai (A.K.); thodoris.siozos@libramli.ai (T.S.); aggelos.pikrakis@libramli.ai (A.P.); 2 School of Information and Communication Technologies, University of Piraeus, 18534 Pireas, Greece; 3 U-Blox AG, 8800 Thalwil, Switzerland; timon.merk@u-blox.com (T.M.); matthias.mahlig@u-blox.com (M.M.); stelios.papaharalabos@u-blox.com (S.P.); peter.karlsson@u-blox.com (P.K.); 4 Movement Disorder and Neuromodulation Unit, Department of Neurology, Charité–Universitätsmedizin Berlin, 10117 Berlin, Germany

**Keywords:** indoor localization, BLE, deep neural networks, angle of arrival

## Abstract

In this paper, we present a novel Deep Neural Network-based indoor localization method that estimates the position of a Bluetooth Low Energy (BLE) transmitter (tag) by using the received signals’ characteristics at multiple Anchor Points (APs). We use the received signal strength indicator (RSSI) value and the in-phase and quadrature-phase (IQ) components of the received BLE signals at a single time instance to simultaneously estimate the angle of arrival (AoA) at all APs. Through supervised learning on simulated data, various machine learning (ML) architectures are trained to perform AoA estimation using varying subsets of anchor points. In the final stage of the system, the estimated AoA values are fed to a positioning engine which uses the least squares (LS) algorithm to estimate the position of the tag. The proposed architectures are trained and rigorously tested on several simulated room scenarios and are shown to achieve a localization accuracy of 70 cm. Moreover, the proposed systems possess generalization capabilities by being robust to modifications in the room’s content or anchors’ configuration. Additionally, some of the proposed architectures have the ability to distribute the computational load over the APs.

## 1. Introduction

Indoor positioning system (IPS) technologies are on the rise over the last decades due to high industrial and domestic demand. Use cases range from indoor navigation (e.g., in hospitals, office buildings, university campuses, airports, etc.), to object localization (e.g., packets in a warehouse, household items, etc.) [1,2,3]. Although global positioning systems have proven to be very efficient in outdoor environments, they fail to perform well indoors due to various factors, mainly the multipath effect, fading and reflections.

For this reason, a variety of indoor positioning systems have been developed, most of them utilizing radio signals such as ultra-wide band (UWB) [4], Wi-Fi [5] or Bluetooth. Among these technologies, Bluetooth Low Energy (BLE) has emerged as an affordable mass market alternative with reduced energy consumption. Even though the impact of fading effects is more notable in BLE when compared to Wi-Fi and UWB, its ease of deployment and compatibility with a multitude of devices render it suitable for numerous short-range communication use cases, including indoor positioning applications [6,7,8,9]. BLE offers direction finding capability through constant tone extension (CTE) packets [10]. More specifically, the angle of arrival (AoA) can be estimated through appropriate switching between subsequent antenna elements and measuring the in-phase and quadrature-phase (IQ) values from the received CTE packets. 

Basic algorithms for AoA estimation involve spectral-based methods, such as the estimation of signal parameters via rotational invariance technique (ESPRIT) [11] and multiple signal classification (MUSIC) [12], by exploiting the IQ-sample covariance matrix. The propagator direct data acquisition (PDDA) algorithm was introduced to lower complexity and computation time by avoiding covariance matrix calculation and matrix inversion [13]. In principle, the AoA for a given anchor is estimated as the peak position in the spatial power spectrum of PDDA. When multiple anchors are employed, the tag position can be estimated with the well-known least squares (LS) algorithm or via triangulation. Some experiments with the PDDA algorithm for BLE AoA estimation and indoor positioning have been reported in [14] showing an average positioning accuracy of less than one meter.

Recently, machine learning (ML) techniques have been utilized for indoor localization problems. For the specific use task of BLE positioning, the k Nearest Neighbors (kNN) algorithm and deep learning models have been employed in fingerprinting approaches by predicting the position based only on RSSI values [15,16,17,18] or on a combination of RSSI and IQ values [19]. In contrast to fingerprinting approaches, deep neural networks may generalize better when the measurement environment undergoes changes with the introduction of additional or different multipath propagation components. Most of the existing approaches that employ neural networks (NNs) for indoor localization, either estimate the position directly [20,21] or via a combination of distance estimation and trilateration [22]. It has to be noted that AoA-based neural network localization approaches exist [23] but have so far been scarce. There are, however, successful applications of NN-based AoA estimation of single APs, using as features IQ values [24], MUSIC generated spatial power spectrum features and exploitation of the temporal domain [25,26] or PDDA generated power spectrum features [27,28]. 

Position estimation jointly based on multiple APs is less susceptible to performance impairments due to non-line-of-sight (NLoS) conditions. In this respect, both Wi-Fi signals (based on Channel State Information CSI) [29] as well as BLE signals (based on RSSI) [21] have been used for localization using deep learning. 

The main contributions of this paper include:

1. ML-powered BLE-based indoor positioning via multiple anchor AoA estimation using both raw IQ values and RSSI estimates is proposed for the first time;
2. A range of novel deep learning architectures, including fully connected multilayer perceptrons and CNNs are proposed and studied regarding their pros and cons;
3. Joint anchor AoA estimates allowing distributed processing across the anchors is studied, to the best of our knowledge, for the first time. In particular, tuples of APs are grouped in smaller models that are then combined to produce the final prediction. Thus, hardware for a single computational expensive unit is replaced by less computationally demanding units distributed among the APs, facilitating embedded implementations;
4. It is shown that deep learning methods yield robust indoor localization generalization, given environmental changes, e.g., different LOS-blocking obstacles and altered AP arrangements. To the best of our knowledge, no other study for indoor localization based on machine learning evaluates the generalizability of its models in different environments than the ones used in training;
5. A data augmentation strategy is introduced that allows for reducing the training data size with minimal impact on performance. The performance improvement potential of joint anchor estimates vs. single anchors is also studied;
6. A novel high spatial resolution dataset with multiple furniture configurations produced with realistic ray-tracing simulations is provided in open access mode.

The rest of this article is organized as follows. Section 2 describes the theoretical foundation of the work, the data processing steps that were taken, the architecture of the developed NN models and the simulation environments. In Section 3, results and comparison of the different methods are presented and discussed, along with some additional experiments regarding model complexity and training set size. Section 4 discusses the major outcomes of this work and, finally, Section 5, presents some concluding remarks.

## 2. Materials and Methods

### 2.1. Theoretical Foundation

As stated in the introduction, BLE offers AoA estimation capabilities, given that specialized hardware is used. This hardware, in our case, consists of a receiver that hosts an L-shaped antenna array. Each AP array receives an analog sinusoidal signal in the form of CTE packets, that is demodulated into IQ values using two sinusoids with the same frequency and a relative phase shift of 90°. Given the sinusoidal signals of adjacent antenna elements, the AoA can be calculated by the following formula:

$$\theta =\cos^{-1}\left(\frac{\psi \lambda }{2\pi d}\right)$$

where *ψ* is the phase difference of the two sinusoids, *λ* is the wavelength and *d* is the distance between adjacent antenna elements.

In addition, an antenna’s measured RSSI can be used to estimate the distance *d* of the tag from the receiver with the following formula,

$$d=10^{\frac{RSSI_{ref}-RSSI}{10n}}$$

where *RSSI_ref_* is the reference *RSSI* value measured at a distance of 1 m and *n* is the attenuation constant. 

As long as the AoA of a number of APs have been estimated, the positioning can be realized through the least squares method. For the simple case of two APs, the position in 3D space can be determined by finding the intersection of the two lines corresponding to the directions of arrival to each AP. However, when there are errors in the estimation of AoA values, an intersection of those lines may not always exist. Moreover, non-existence of an intersection is even more likely when there are more than two anchors making the system overdetermined. This issue can be avoided if we solve for the least squares error solution of the system formed by the equations:

(1) $$\begin{array}{c} \beta_{i}x-\alpha_{i}y=\beta_{i}x_{a,i}-\alpha_{i}y_{a,i}\\ \gamma_{i}x-\alpha_{i}z=\gamma_{i}x_{a,i}-\alpha_{i}z_{a,i} \end{array},\;\;for\;i=1,2,3,4$$

(2) $$\{\begin{array}{l} \;\;\alpha_{i}=\cos\varphi_{i}cos\theta_{i}\\ \;\;\beta_{i}=\sin\varphi_{i}cos\theta_{i}\\ \;\;\gamma_{i}=\sin\theta_{i} \end{array}$$

Angles $\varphi_{i}$ and $\theta_{i}$ correspond to the estimated azimuth and elevation angles for AP_i_, where $\theta_{i}\;$ is the complementary of the polar angle of the spherical coordinate system and $a_{i}=\left[x_{ai},\;y_{ai},\;z_{ai}\right]$ is the position of AP_i_. The pairs of equations that form the system correspond to pairs of planes whose intersection is the direction of arrival line. Alternatives, e.g., the one in [30], could also be used for the AoA to location conversion. 

### 2.2. System Setup and Data Preprocessing

In this work, an arbitrary number of APs can be used, say k, that collect the RSSI and IQ value measurements from all their antenna elements, say p. Moreover, several BLE channels, say r, are considered and for each antenna-channel set we exploit only the measurements that correspond to the polarization with the highest RSSI. These yields to p IQ value pairs and one RSSI value per AP per channel, i.e., 2p+1 values total for each AP’s channel. An important processing step that we adopt is to remove the absolute phase information of the IQ values while keeping the phase differences intact. Consider a stationary tag and the p vectors $\left[I_{i},\;Q_{i}\right]$ measured by the antennas of a single AP in any channel. Vector phases will have varying values depending on the time of flight and the transmission time phase. By expressing the p measured phases in relation to the phase of the first antenna, using the formula $\varphi_{i}^{\prime }=\varphi_{i}-\varphi_{0}$, we can remove the transmission time phase information and keep only the phase differences, which are the most useful for AoA estimation. By using this transformation of the phases, the quadrature-phase component of the first antenna element is always zero, hence the corresponding feature is removed resulting into a feature vector of length 2p−1 plus 1 for the RSSI value. Finally, the RSSI values of all the channels in each AP are standardized to have zero mean and match the variance of the IQ data of the certain anchor. Hereafter, for demonstration purposes, we consider the case of 4 APs, where each one comprises 5 antenna elements. We also focus on the three advertising channels that BLE operates on, i.e., channels 37, 38 and 39 with respective transmitting frequencies of 2402, 2426 and 2480 MHz. For convenience, although the proposed novel NN architectures that we will present next, correspond to this specific case, they are straightforwardly extendable to other AP numbers, antennal elements, and number of channels.

### 2.3. NN Model Architectures

Five different neural network architectures were designed to investigate different aspects of the problem at hand. All the models aim to estimate the AoA values of all APs, which are then used to compute the location of the tag in the xy-plane via LS estimation, as it was discussed in Section 2.1. This approach resembles the common muti-anchor indoor localization processing pipeline.

**Figure 1:**
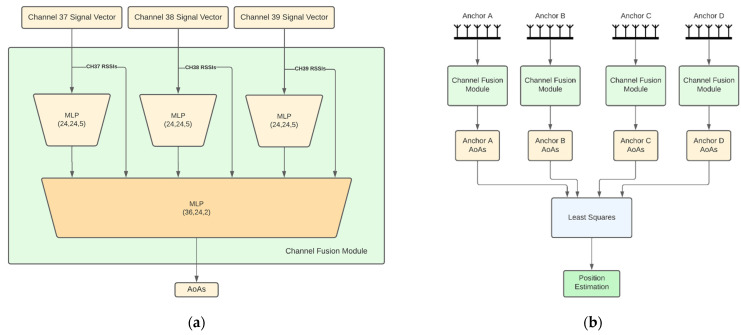
(**a**) Channel fusion module; (**b**) independent AP architecture. Layer sizes for each MLP are shown in parentheses.

**Figure 2:**
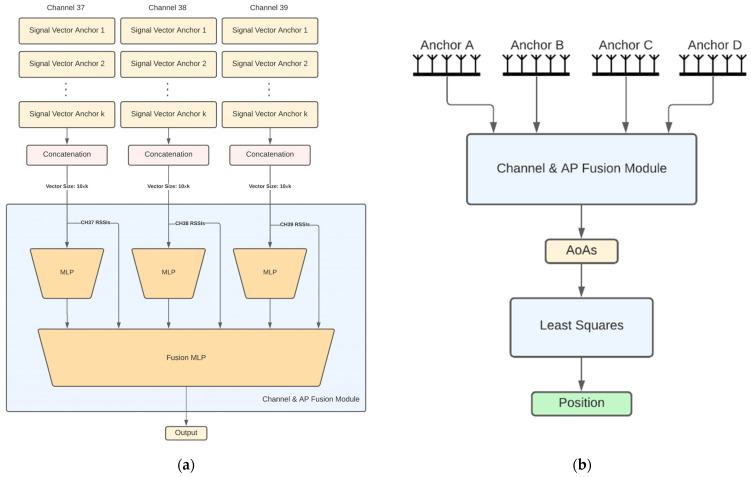
(**a**) Channel and AP fusion module; (**b**) Fully joint AP architecture.

**Figure 3:**
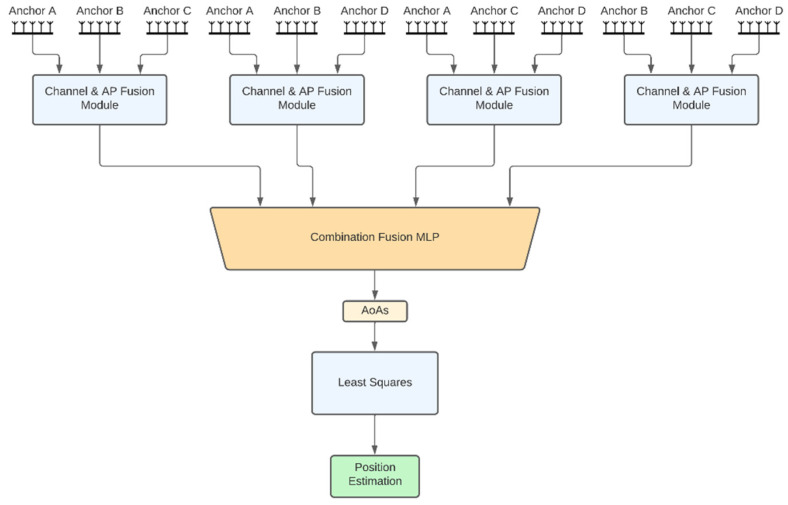
Triplets of APs architecture.

**Figure 4:**
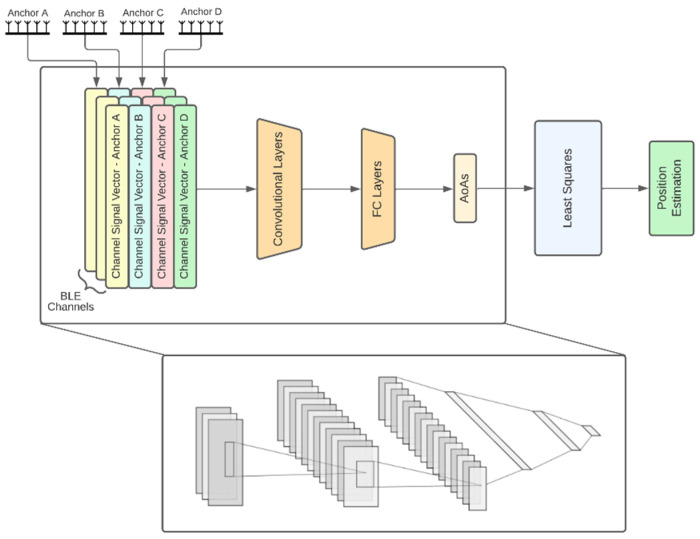
Architecture of CNN-based joint APs.

The description of each architecture follows:

1. Independent APs: In this architecture (Figure 1b), each AP has its own model for AoA estimates and the models are trained independently from each other. Each AP model input consists of three feature vectors of length 10, one per channel. Each vector consists of 9 IQ values and the channel RSSI value. This vector is hereafter referred to as *channel signal vector*. Each channel signal vector is fed to a different 3-layer neural network that produces a 5-dimensional latent representation. The 3 latent representations are then concatenated together with the RSSIs and fed to a 3-layer channel fusion MLP, which outputs the 2 angular directions (azimuth and elevation) of the AoA. In the sequel, we refer to this module as the *channel fusion module* (Figure 1a). Note that this architecture along with the ones presented later on, are directly extendable to more than 3 BLE channels and are not bound to the configuration that is chosen here for demonstration purposes. An advantage of the independent APs architecture is that the computational requirements are distributed across the anchors. However, this architecture does not exploit the fact that the APs are placed in fixed positions in a specific room, so the signal received by an AP from a tag placed anywhere in the room also conveys information about the AoA of this signal to the rest of the APs. This shortcoming is addressed by joint AP architectures discussed next.
2. Fully joint APs: This architecture aims to jointly estimate the AoA values of all APs using the respective received signals. Before going into the details for this architecture, let us first describe the *channel and AP fusion module* shown in Figure 2a, which is the main building block of this architecture and the following ones. This NN module first computes a latent representation for each channel and for a set of APs of size *k*, where *k* is a hyperparameter. To this end, the channel signal vectors of all anchors are concatenated per channel and fed as input to 3 different 3-layer MLPs with layer sizes 60, 40 and 12. Then the outputs of these MLPs are concatenated and, together with all the channel RSSIs, are fed into another 2-layer fusion MLP with layers of size 64 and 8, respectively. The fully joint AP architecture, shown in Figure 2b, is essentially the channel and AP fusion module. Accordingly, the fusion MLP output layer has 8 nodes to produce the final AoA estimates for all APs (2 angular directions per AoA × 4 APs). An advantage of this architecture compared to the independent AP one is the performance improvement, as will also be discussed in Section 3. A disadvantage is the lack of the distributed processing capability, which means that a powerful enough central computing unit is required to collect all signals and run the model.
3. Tuples of APs: This architecture aims to combine the best features of the two aforementioned architectures, namely, high performance and distributed computing potential. The idea is to jointly tackle *k*-combinations with repetition. Taking for example *k =* 3, exemplary possible AP combinations are ABC, ABD, ACD and BCD. Then, the corresponding triplets of APs architecture is shown in Figure 3, where the channel signal vectors of the four distinct triplets feed corresponding channel and AP fusion modules. In this case the three different MLPs of the channel and AP fusion module consist of 2-layers with sizes 24 and 9 and the Fusion MLP consists of 2-layers as well, but this time with sizes 27 and 12. Subsequently, the latent representations from the outputs of these models are concatenated and fed to a final 3-layer combination fusion MLP, with sizes 32, 16 and 8. The final output are the 8 AoA values for all 4 APs. As shown in Section 3, the performance of this architecture is similar, if not better, to that of the fully joint setup. In addition, the computational complexity can be distributed across the four APs if edge computing units embedded in the APs perform the computations of each channel and AP fusion module in parallel. The computations for the MLP that performs the final fusion along with the LS-based estimates of the positioning, still need to be performed subsequently. Similar to the triplets of APs architecture, one can also define the pairs of APs architecture.
4. CNN-based joint APs: This is an alternative joint architecture that operates on measurements taken from all four APs simultaneously using convolutional neural networks (CNNs), as shown in Figure 4. A major difference compared to the architecture of fully joint APs is that the channel and APs fusion module has been replaced by convolutional layers. To achieve this, the channel signal vectors are rearranged to form a 2D image-like representation of size 10 × 4 × 3, where the dimensions correspond to the APs, the channel signal vectors and the BLE channels, respectively. The 2 convolutional layers use kernels of size 4 × 1 and 3 × 2, respectively, and are followed by 3 fully connected layers, as shown in Figure 4.

For more details, concerning the model architectures and data processing, please refer to the Supplementary Materials Section which links to the project’s github repository.

### 2.4. Simulation Environment

The Altair Feko WinProp software has been used in order to simulate ray-tracing propagation data in a pre-defined area/indoor scenario. After running this software, the propagation area results (e.g., field strength, path loss, delay spread, angular spread, etc.) are stored for AoA and distance estimation. Our core simulation environment is a room with dimensions 14 m × 7 m including several furniture configurations. Four horizontal facing APs are placed in the corners of the room at 2.5 m height and at 45 degrees azimuth rotation pointing towards the center of the room. The elevation angle of all APs is 45 degrees pointing downwards. Three transmitting frequencies are simulated, i.e., 2402, 2426 and 2480 MHz, corresponding to the three advertising BLE channels (numbered 37, 38 and 39, respectively) and two antenna polarizations (omni-directional), i.e., horizontal and vertical. The tag is positioned at a fixed height of 1.5 m (z-dimension) and 2450 signal samples are collected per configuration, evenly distributed across the room. This corresponds to a spatial sampling resolution of 20 cm across x and y dimensions. The aim of such a dense sampling grid is to thoroughly evaluate the spatial generalization of the proposed methods. In particular, the models are trained using a small fraction of these data-points, e.g., 140 points, and their performance is evaluated on the remaining locations that have not been used for model training. 

**Figure 5:**
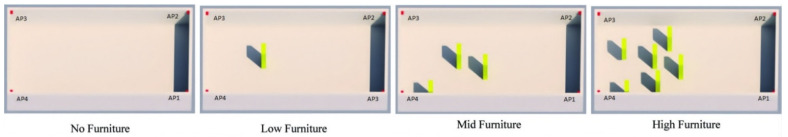
Visualization of the four room configurations.

Regarding LoS blocking furniture configurations, four different scenarios are considered which are illustrated in Figure 5. These are:

1. The no LoS-blocking furniture case (referred to as “No Furniture”);
2. Having one piece of LoS-blocking furniture, covering 0.5% of the room’s area (referred to as “Low Furniture”);
3. Having three pieces of LoS-blocking furniture, covering 1.5% of the room’s area (referred to as “Mid Furniture”);
4. Having six pieces of LoS-blocking furniture, covering 3% of the room’s area (referred to as “High Furniture”).

**Figure 6:**
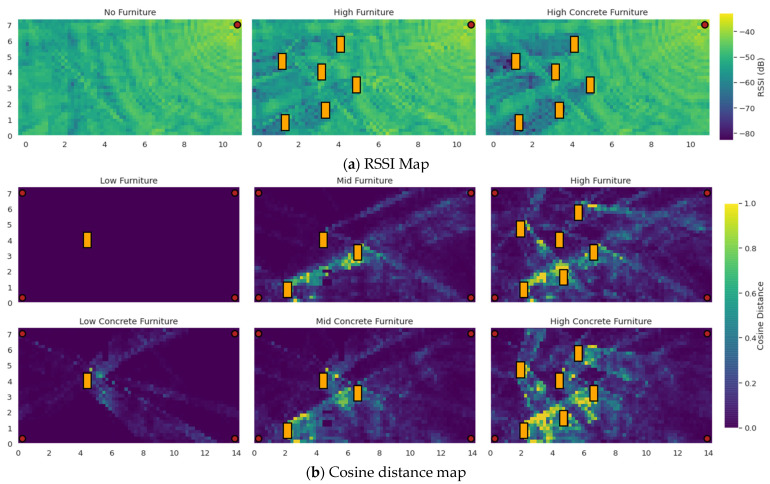
(**a**) Measured RSSI values by a single AP for 3 different rooms configurations and (**b**) cosine distance of IQ features between the low furniture room configuration and the rest of the furniture configurations.

Note that in all cases there exist extra furniture of low height that is not LoS-blocking, which is not shown in the Figures. Moreover, although the percentage coverage of the furniture might look small, it does not reflect the actual LOS-blocking potential of the furniture due to the fact that each piece of furniture is relatively thin but wide. This point is later further discussed along with the furniture impact study of Figure 6. In every configuration, the furniture is distributed in a non-uniform way, to examine the behavior of our models in furniture dense and furniture free areas. Regarding material, all wooden and all concrete furniture have been simulated with varying reflection parameters. The above furniture quantity and material combinations sum up to seven distinct datasets. Moreover, for the No Furniture configuration, we have also generated data after applying a clockwise rotation of 5 degrees or after applying a translation of 10 cm to each AP. This will allow us to evaluate the ability of the models to deal with moderate AP displacements.

The furniture distribution was chosen to enable the performance study of both furniture dense and furniture free areas. Therefore, all the furniture occupies the left half of the room. Regarding furniture size, we realized that by placing furniture of relatively small area size close together, it generated particularly hard areas to tackle, usually in between the furniture. To better understand the effect of furniture placement and configurations, we provide RSSI maps (Figure 6a) and cosine distance maps (Figure 6b) for some of the used room configurations to demonstrate that an appropriate level of differentiation exists between them. This further reinforces our conclusions concerning the generalization capability of the proposed methods, presented in Section 3.4.

The maps of Figure 6b depict the cosine distance per point for the IQ features received by all APs in the low wooden furniture case and those received in the mid wooden, high wooden, low concrete, mid concrete and high concrete furniture cases. More specifically, the vector used to calculate the cosine distance between any location of the low wooden furniture room and the same location of the rest of the rooms is formed by concatenating the channel signal vectors for all 3 channels and all 4 APs. From two such vectors, say x and y, the cosine distance can be calculated by the following expression:

$$CD\left(x,y\right)=1-\frac{xy}{\left\|x\|\|y\right\|}$$

Observe that the addition of LOS-blocking furniture results into a significant alteration of the IQ features in large areas of the room (locations with cosine distance close to one correspond to IQ features completely uncorrelated with the original room configuration’s IQ features).

In total, 7 different datasets are used, 5 for the described room configurations and 2 for the described AP configurations. Each dataset contains IQ and RSSI measurement data from all 4 APs and for the 3 advertising channels resulting in 4×3×10=120 features per point location. For more details, concerning the dataset and ways to access it refer to the data availability statement at the end of the article.

## 3. Results

A set of experiments has been conducted in order to assess the performance of the proposed architectures. The main performance metric that we use is the Mean Euclidean Distance Error (MEDE), which is the mean distance between each predicted tag location ${\hat{P}}_{i}$ and the corresponding true location $P_{i}$.

(3) $$\mathrm{MEDE}\left(P,\;\hat{P}\right)=\frac{1}{N}\overset{N}{\underset{i=1}{\sum }}d\left(P_{i},{\hat{P}}_{i}\right)=\frac{1}{N}\overset{N}{\underset{i=1}{\sum }}\sqrt{{\left(X_{i}-{\hat{X}}_{i}\right)}^{2}+{\left(Y_{i}-{\hat{Y}}_{i}\right)}^{2}+{\left(Z_{i}-{\hat{Z}}_{i}\right)}^{2}}$$

We have also measured the performance of the models regarding the AoA estimates, in terms of Mean Absolute Error (MAE), which is defined as the mean absolute difference between predicted and true values, i.e.,

(4) $$MAE\left(A,\hat{A}\right)=\frac{1}{N}\overset{N}{\underset{i=1}{\sum }}\left|A_{i}-{\hat{A}}_{i}\right|$$

In the above formulas, *N* is the number of test location points, $\left({Χ}_{i},\;Y_{i},\;Z_{i}\right)$ is the true position for point *i*, and $\left({\hat{Χ}}_{i},\;{\hat{Y}}_{i},\;{\hat{Z}}_{i}\right)$ is the predicted position for point *i*. MAE is calculated separately for the azimuth and elevation components of the AoA.

Machine learning models for indoor localization may suffer from low generalization potential, i.e., the model may have difficulties to perform well with data cases never seen during training. In our study, we examined several generalization aspects, such as the spatial generalization potential of the models, i.e., to perform well in locations never seen during training and the generalization of different furniture configurations, e.g., furniture density and furniture material. Moreover, we investigated the robustness of the models to moderate dispositioning of the APs.

The performance of the proposed NN-based architectures is compared against the PDDA-based estimations. AoA estimation for a single AP, given the power spectrums generated by PDDA, is as straightforward as determining the angle corresponding to the maximum power spectrum value. However, a different power spectrum is generated for each channel and polarization combination, resulting in 6 spectra in total. It was observed that the best results are achieved using the spectrum that results from multiplying the spectrum corresponding to the highest RSSI polarization for each channel.

### 3.1. Performance on a Fixed Environment

The aim of this experiment is to study the performance of the proposed architectures on a fixed environment, that is, on the same configuration that was used for training. To achieve this, we train with the data corresponding to a small number of locations across the room and compute the performance on the data of the rest of the locations of the same room. This performance evaluation approach has so far been the gold-standard in most of the ML-based and fingerprinting techniques. We focus on the low and high furniture configurations and, for the sake of consistency, among configurations, all locations that overlap with the furniture of the high furniture case are ignored, leading to 2293 available locations in each room. For training, we used 140 locations that belong to a uniform grid with step size of 1.2 m for the *x*-axis and 0.6 m for the *y*-axis. For validation, another 30 points were picked that belong to a grid with step sizes of 2.4 m for the *x*-axis and 1.2 m for the *y*-axis. The rest of the points, i.e., over 90% of the total datapoints have been used for testing. The distribution of the train, validation, and test locations is studied further in Section 3.5. Each point sample consists of a total of 4×3×10 features as explained in Section 2.2. The scale parameters for the standardization of the RSSI features are acquired from the training data only and are then used to scale the test data during inference time to avoid circular test data leakage within the training preprocessing pipeline.

To further improve the performance of the proposed NN architectures, we also augment the available data. Our aim is to artificially create new realistic NLoS scenarios that are not present in the available data and that might, for example, arise from alternative furniture configurations. In particular, we are getting 30 augmented signals for each one of the training points by stochastically reducing the amplitude of the IQ values and the RSSI value of the anchors. For each augmentation instance, there is a 70% chance of deteriorating the signal for just one AP, 20% chance for simultaneously deteriorating the signal of two APs and 10% chance for deteriorating the signal of three APs. Moreover, the amplitude is randomly reduced by dividing with a number that is sampled uniformly in the interval (1.1,  5). The amplitude reduction is the same for all the antenna elements of each anchor for a single augmentation instance. The above augmentation procedure is applied to each BLE channel separately and the corresponding channel RSSI values are reduced accordingly. 

Next, we compare the performance of all proposed NN architectures when trained both with the original and the augmented data, against the PDDA performance. It should be noted that all the NN architectures were designed to have around 20K parameters in total in order to guarantee that they are similarly computationally complex. Moreover, in order to be fair to the PDDA method, only the estimates of azimuth angles are considered, since all data points correspond to the same horizontal plane, having a fixed height of 1.5 m. When both the azimuth and elevation angles are considered, PDDA’s performance exhibits a degradation of about 40%, whereas the performance of the suggested NN architectures is not affected. Consequently, we ignore the $\hat{Z}$ estimates during the calculation of MEDE. The results for the two aforementioned rooms in MEDE (in meters) are shown in Table 1. 

It can be observed that in all cases the proposed models outperform PDDA both in MEDE and in standard deviation, with the models considering jointly all or subsets of the APs performing better than the independent Anchor architecture. Moreover, data augmentation leads to notable improvements in the performance of all NN models with the joint models exhibiting larger improvements against the independent ones. In general, the CNN-based joint architecture exhibits the best performance in all scenarios. The above results are confirmed by the Azimuth AoA MAEs of each AP presented in Table 2. As an indicative example, we focus on a hard configuration, namely the high furniture, while also employing the data augmentation scheme described above. Note that the performance of the NN-based approaches is more consistent across APs compared to the PDDA one. For example, although PDDA has a competitive MAE of 5.80 in AP1, its performance degrades significantly in AP2. On the contrary, all joint methods exhibit similar performance in these two APs, since the estimates of a hard to tackle AP are assisted by the better conditioned signals received to the rest of the APs. 

### 3.2. Generalization Performance on Environment Configurations Not Seen during Training

In the previous evaluation setup, the NN models had a considerable advantage over the PDDA method, as they were tested in the room configuration that was used for training. This has limited practical use, since it is highly likely that the mobile elements of a room will change with time. Therefore, this experiment aims to test the robustness of the proposed methods when room configuration changes. To this end, the models are trained with 140 location points from a certain room and are evaluated on the remaining rooms, in location points that were not used for training. Moreover, the data is augmented since this approach was shown to clearly enhance performance.

**Figure 7:**
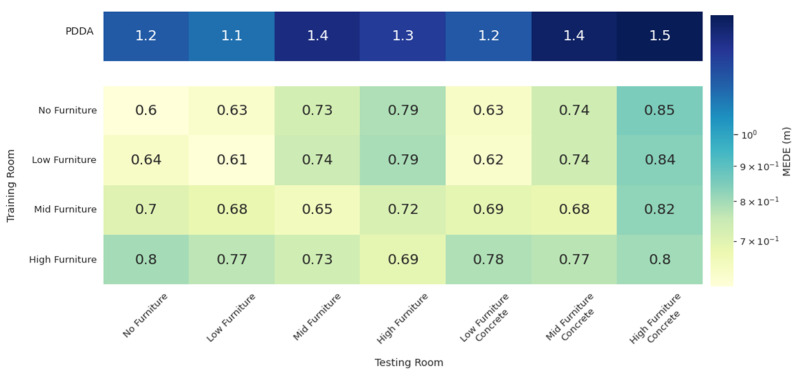
MEDE of the CNN-based joint model on different rooms. PDDA methods performance in each room is included for reference.

**Figure 8:**
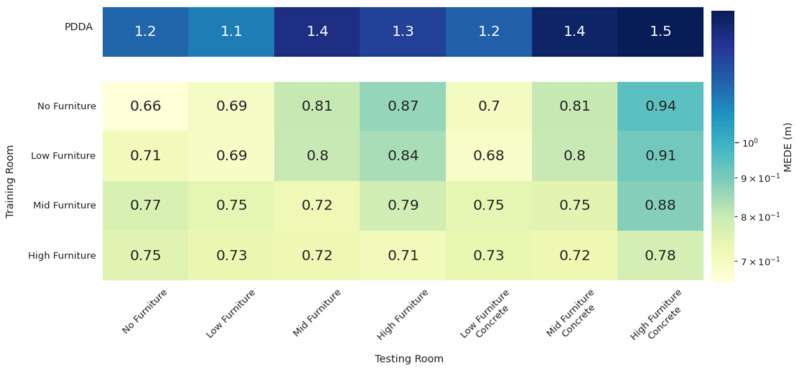
MEDE of the pairs of APs model on different rooms. PDDA methods performance in each room is included for reference.

The results for the architectures of CNN-based joint APs and Pairs of APs are shown in Figure 7 and Figure 8, respectively. For reference purposes, the top row of the tables shows the PDDA performance for all room configurations, which is the same in both cases. The other table rows indicate the room in which the models have been trained, whereas columns indicate the room in which the models were evaluated. For example, the second row corresponds to models trained in the low furniture configuration and tested in the no, low, mid and high furniture configurations as well as in the low, mid and high furniture configurations with the furniture being made of concrete rather than wood. 

Apparently, even though some performance degradation is introduced by furniture height and material, all NN models perform well in all situations and can thus be considered to exhibit some generalization capability. Moreover, generalization is improved when the models are trained in more demanding conditions, as in the high furniture case. Regarding performance, the worst scenario in both models is when training in the no furniture case and testing in the high furniture concrete, since this leads to blocking more LoS signal paths and adding stronger reflections. The MEDE achieved is 0.85 m and 0.95 m for the CNN-based and the pair of APs architectures, respectively. Both are significantly improved over the PDDA that achieves 1.5 m error in the specific room configuration.

### 3.3. Study of the Error Spatial Distribution

**Figure 9:**
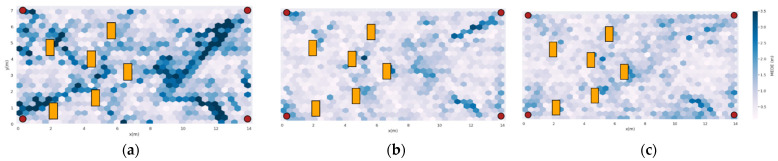
Spatial distribution of Euclidean error on the high furniture room of (**a**) PDDA; (**b**) CNN-based joint model; (**c**) pairs of APs Model.

To complete the previous study, we now examine the performance of some of the methods over the room space. Figure 9a shows the performance of PDDA and Figure 9b,c shows the performance of the CNN-based joint architecture and the pair of APs architecture, respectively, when they are both trained in a different room configuration, namely the low furniture one. 

PDDA exhibits large performance variation across the room, which was already expected given the large standard deviation of the error. On the contrary, the pair of APs architecture exhibits the best spatial generalization capability, yielding only a few micro-cells in the blue-to-dark-blue color spectrum. Note that spatial generalization capability is not directly linked to the overall performance, because, as it has been shown in Table 1, the overall performance of CNNs is better than the pairs of APs one.

An additional experiment was conducted to assess and compare performance between furniture-dense and furniture-free areas of the room, i.e., the left-half and the right-half part of the room, respectively. In this experiment, the results of which are presented in Table 3, the models were trained again on the low furniture room and were tested on the high furniture room. The furniture-dense half seems to be the best performing one for most of the models. We believe that this happens because the LoS of the two left-most AP is heavily blocked by the nearby furniture, which results in high AoA estimation errors for those two APs in the right half of the room (i.e., the furniture free part). Moreover, the AoA errors are proportional to the distance from the APs, further resulting in higher localization errors in the right half of the room. The independent model, the fully joint model and the PDDA algorithm seem to be resilient to this problem, making them more suitable to non-uniform furniture distributions. In any case, it apparent that even though the furniture is not distributed across all of the room, its presence affects the performance in any location.

### 3.4. Study of Model Generalization against Moderate AP Displacements

Another critical aspect regarding practical implementation and use of NN-based modes, is their generalization capability regarding moderate displacements of the APs, that might happen accidentally. To test this scenario, we evaluated the models in the case that the test data correspond to altered APs configurations. They have all been rotated by 5° horizontally or they have been displaced by 10 cm. This choice was made to simulate real-life conditions in which the state of some of the APs may change in some extent. 

**Figure 10:**
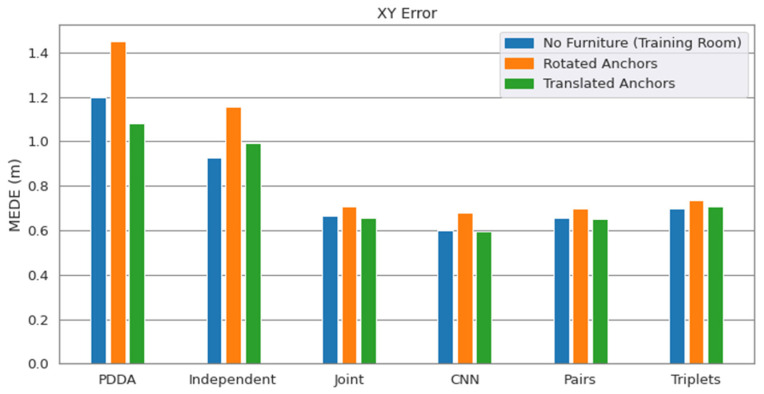
Performance degradation when the anchor points are rotated by 5° or translated by 10 cm.

To focus on the study of the above disturbances, the no furniture room is adopted for training and testing. Note that the system is not aware of the changes in the APs’ configuration, meaning that the original AP positions and orientations are used for the AoA to position estimation through LS. In the results presented on Figure 10, we observe a great generalization potential, with the joint approaches being once again more robust than the independent APs architecture and PDDA. Apparently, the new anchor positions lead to somewhat better conditions regarding LoS components, which is reflected in improved PDDA performance. However, AP rotation seems to be much more of an issue than the slight AP translation in space.

### 3.5. Impact of Model Size and Training Dataset Size

**Figure 11:**
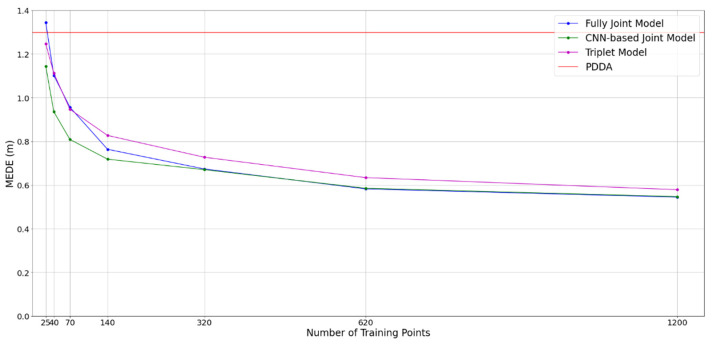
MEDE of joint model across all rooms with different number of training points.

All the NN-based simulations, that have been presented so far, correspond to 140 training location points and with the overall number of parameters (multiplications) being approximately 20K. In this subsection, we investigate how model performance is affected by the number of training points and complexity reduction. To have an overview of all the simulation cases, all performance results correspond to an average of the MEDEs of the grids shown in Figure 7 and Figure 8. We evaluated the performance of the triplets of APs and fully joint and CNN-based architectures in Figure 11 with varying numbers of training data points, evenly distributed across the room. We observe that going from 140 to 1200 data points, an improvement of about 0.2 m can be achieved. However, the improvement rate degrades with the amount of training data, so there is not a strong reason to go beyond 320 training data points, given data acquisition challenges. On the contrary, we observe that the models keep performing better than PDDA, even when only 40 training data points are used for training. 

**Figure 12:**
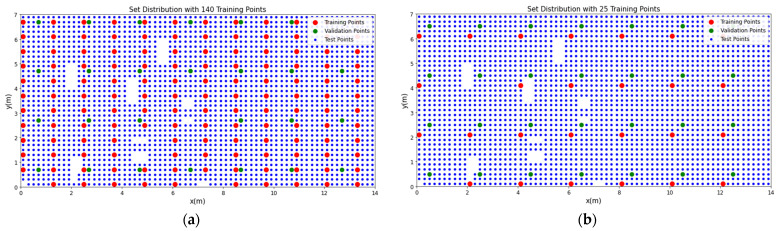
Training (red dots), validation (green dots) and test locations (blue dots) for training set of size (**a**) 140 and (**b**) 25.

We can see the distributions of the different sets for 140 training data points and for 25 training data points in Figure 12a,b, respectively. The empty spaces between the data points correspond either to furniture, or to the absence of data points. We notice that 25 training points can provide adequate coverage of the room which can be effectively exploited by the CNN-based joint architecture.

**Figure 13:**
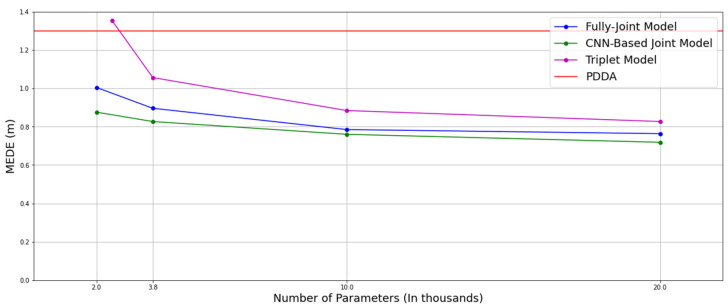
MEDE of fully joint model and CNN-based joint model across all rooms with number of parameters.

Figure 13 shows the relationship between performance and model size. We observe minor performance degradation when the overall number of parameters falls to half, i.e., to 10K, and that one can see considerable improvement over PDDA performances even with 2K parameters. Note here that the distributed architecture shown, i.e., the triplets of APs architecture exhibits a severe degradation below the 3.8K parameters. However, the overall complexity per triplet of anchors is very limited, since it comprises 600 parameters and there are 4 triplet computations, that can be distributed across the 4 anchors. Then, the joint processing of all latent representations resulting from the triplets is achieved by the fusion model comprising 100 parameters only.

## 4. Discussion

In this work, several novel NN architectures are proposed for the problem of indoor localization via AoA estimates based on multi-anchor, multi-channel BLE signals. To thoroughly assess model performance and behavior under well-controlled environments and known ground truths, we have implemented a multi-scenario simulation dataset using accurate and realistic ray-tracing propagation modeling. Such a dataset is highly desirable because it allows for the evaluation of any developed ML model with respect to critical generalization aspects, including spatial generalization, generalizations regarding different furniture configurations and material and generalizations regarding mild displacements of the installed APs.

Emphasis has been given to joint anchor architectures, where the data received from all or from subsets of anchors is used as input to ML models that jointly estimate the AoA of the corresponding anchors. The best localization performance is achieved by the models that exploit simultaneously the full set of anchors, namely, the fully joint and the CNN-based joint APs architectures, with the latter appearing to be more robust to both reduced number of training points and lower complexity configurations. On the other hand, the independent APs and the tuples of APs architectures, have the potential of distributed processing across anchor point. Additionally, the pairs of APs architecture appear to offer a slightly better spatial generalization potential compared to the other ones, including the CNN-based joint APs architecture. Overall, all the proposed approaches appear to generalize well into several changes of the simulation environments, and they are also well suited to data augmentation strategies. Moreover, all the methods significantly outperform PDDA, which serves as the major benchmark. PDDA performance is consistently worse by over 50% compared to that of the proposed joint APs architectures even when the models are trained in furniture configurations and materials different than those tested. To summarize the pros and cons for all derived NN model architectures, we present Table 4, that contains the main characteristics as well as the advantages and the disadvantages of each architecture.

Beyond the indoors localization task, all the suggested models can also be used in any applications where AoA estimates are needed.

## 5. Conclusions

In this paper, several NN architectures for indoors localization and/or AoA estimation based on IQ and RSSI values as inputs have been implemented and studied in realistic simulation environments. The developed models are robust against modifications of room furniture configurations and materials and against moderate displacements of the APs exhibiting a high generalizability potential. 

Future directions of research include adjustments to the suggested architectures to include trainable layers that will automatically weight the APs’ contribution depending on their signal quality, offering a larger potential to be explained. Moreover, the proposed methods will be evaluated in real-word data and scenarios. Finally, we intend to study the option to pretrain the models with loads of data produced by a diverse set of realistic simulation environments and then fine-tune in actual real-life scenarios.

## Figures and Tables

**Table 1:** MEDE ± standard deviation comparison of each model and PDDA when evaluated in 2123 spatially distributed locations that have not participated in model training of the low furniture and the high furniture rooms.

Model	MEDE in Low Furniture (Meters)		MEDE in High Furniture (Meters)	
	Non-Augmented	Augmented	Non-Augmented	Augmented
Independent	1.03 ± 0.78	0.96 ± 0.74	1.10 ± 0.86	1.08 ± 0.86
Fully Joint	0.75 ± 0.53	0.65 ± 0.47	0.86 ± 0.60	0.75 ± 0.52
Triplets of APs	0.82 ± 0.52	0.70 ± 0.48	0.96 ± 0.60	0.84 ± 0.57
Pairs of APs	0.76 ± 0.50	0.69 ± 0.48	0.91 ± 0.57	0.71 ± 0.48
CNN-based Joint	0.76 ± 0.49	0.61 ± 0.45	0.87 ± 0.53	0.69 ± 0.50
PDDA	1.14 ± 0.95	1.14 ± 0.95	1.30 ± 1.14	1.30 ± 1.14

**Table 2:** Comparison of each model’s AoA MAE when evaluating in a single room configuration.

Model	MAE in High Furniture (°)			
	AP_1_	AP_2_	AP_3_	AP_4_
Independent	5.64 ± 8.33	6.87 ± 7.29	6.27 ± 8.22	6.2 ± 9.37
Fully Joint	4.18 ± 7.80	4.44 ± 5.51	3.89 ± 4.79	4.38 ± 7.06
Triplets of APs	4.54 ± 6.96	4.62 ± 6.29	4.26 ± 5.34	4.82 ± 6.92
Pairs of APs	4.02 ± 7.35	4.25 ± 5.48	3.99 ± 5.15	4.03 ± 6.50
CNN-based Joint	4.03 ± 6.76	3.89 ± 4.86	3.50 ± 4.8	4.31 ± 6.98
PDDA	5.80 ± 9.14	8.38 ± 10.34	5.86 ± 9.57	7.40 ± 11.26

**Table 3:** Comparison of each model’s MEDE on the left and right halves of the high furniture room.

Model	MEDE (m)	
	Left Half (Furniture Dense)	Right Half (Furniture Free)
Independent	1.12	1.13
Fully Joint	0.80	0.79
Triplets of APs	0.79	0.88
Pairs of APs	0.80	0.88
CNN-based Joint	0.73	0.85
PDDA	1.23	1.36

**Table 4:** Advantages and disadvantages of the different model architectures.

Model	Description	Advantages	Disadvantages
Independent	AoA computed independently by each AP	Simple implementation, Distributed computation	Lower accuracy compared to the rest of the models
Fully Joint	Joint estimation of all AoAs by a single ML model	High accuracy	All raw data need to be transferred to a central unit for computation
Tuples of APs	Joint estimation of AoAs by forming groups of APs	High accuracy, Distributed computation	Performance degrades faster compared to the rest of the models when lower complexity NN configurations are adopted.
CNN-based Joint	Joint estimation of AoAs by a single CNN	Highest accuracy, Most robust approach to complexity reduction and to smaller training size	All raw data need to be transferred to a central unit for computation

## Data Availability

The simulation data used for training and testing is hosted publicly at https://doi.org/10.5281/zenodo.6303665 (accessed on 28 February 2022).

## Supplementary Materials

Python scripts and notebooks with implementations of the ML architectures as well as the data processing pipelines can be found in https://github.com/LIBRA-AI-Tech/sensors-positioning (accessed on 13 March 2022).

## Abbreviations

The following abbreviations are used in the manuscript:

BLE	Bluetooth low energy
Τag	Transmitter
AP	Anchor point
RSSI	Received signal strength indicator
IQ	In-phase and quadrature-phase
AoA	Angle of arrival
ML	Machine learning
LS	Least squares
IPS	Indoor positioning system
UWB	Ultra-wide band
CTE	Constant tone extension
ESPRIT	Estimation of signal parameters via rotational invariance technique
MUSIC	Multiple signal classification
PDDA	Propagator direct data acquisition
Knn	k Nearest Neighbors
NN	Neural network
LoS	Line of sight
NLoS	No line of sight
CNN	Convolutional neural network
MEDE	Mean Euclidean distance error
MAE	Mean Absolute Error
